# Psychological operationisms at Harvard: Skinner, Boring, and Stevens

**DOI:** 10.1002/jhbs.22071

**Published:** 2020-12-10

**Authors:** Sander Verhaegh

**Affiliations:** ^1^ Department of Philosophy Tilburg University Tilburg The Netherlands

**Keywords:** behaviorism, B. F. Skinner, Douglas McGregor, E. G. Boring, operationism, P. W. Bridgman, S. S. Stevens

## Abstract

Contemporary discussions about operational definition often hark back to Stanley S. Stevens' classic papers on psychological operationism. Still, he was far from the only psychologist to call for conceptual hygiene. Some of Stevens' direct colleagues at Harvard—most notably B. F. Skinner and E. G. Boring—were also actively applying Bridgman's conceptual strictures to the study of mind and behavior. In this paper, I shed new light on the history of operationism by reconstructing the Harvard debates about operational definition in the years before Stevens published his seminal articles. Building on a large set of archival evidence from the Harvard University Archives, I argue that we can get a more complete understanding of Stevens' contributions if we better grasp the operationisms of his former teachers and direct colleagues at Harvard's Department of Philosophy and Psychology.

## 1 INTRODUCTION

In 1935, Stanley S. Stevens published two articles in which he urged for a “revolution that will put an end to the possibility of revolutions” in psychology. Building on Bridgman's methodological prescriptions for physicists, Stevens (1935a, p. 323) argued that all psychological concepts need to be strictly defined in terms of public and repeatable operations. If the study of mind and behavior is to be taken seriously as rigorous science, Stevens argued, psychologists have to make sure that they are not talking at cross purposes when they are discussing their theories about “experience,” “consciousness,” and “sensation”—they have to make sure that their concepts are “operationally defined.”

Stevens' call for conceptual rigor was spectacularly successful. Within a few years after the appearance of his papers, Sigmund Koch (1992, p. 269) remembers, “virtually everyone in psychology […] was some kind of operationist. It was as if the adjective “operational” had become cemented to the noun ‘definition'”. Even today, most textbooks in psychology teach students that definitions ought to be operational, arguing that “providing an operational definition of variables” is “a hallmark of well‐conducted research” (Carlson et al., 2010, p. 55). Although few contemporary psychologists accept the most radical implications of operation*ism*—e.g., the view “that the meaning of a psychological concept is *nothing more* […] than the set of operations by which the conceptual entity is observed” (Stevens, 1946, my emphasis)—the view that our variables require operational definitions is as common today as it was 80 years ago.

In view of Stevens' success in spreading the operationist message, it is small wonder that his papers are still widely cited in contemporary discussions about operational definition. Still, he was far from the only psychologist to call for conceptual hygiene. Some of Stevens' direct colleagues at Harvard—most notably B. F. Skinner and E. G. Boring—were also actively applying Bridgman's conceptual strictures to the study of mind and behavior. Skinner used Bridgman's perspective to rework the foundations of behaviorism, whereas Boring and his student Douglas McGregor (1935) wrote a paper in which they developed an operationist perspective to redefine psychophysics as a natural science. Outside Harvard, too, scholars were actively developing operationist views about the nature of psychological concepts (e.g., Hull, 1943; Tolman, 1936). In addition, like‐minded movements also started to gain traction in the philosophy of science (e.g., Blumberg and Feigl, 1931; Campbell, 1928).^1^

Existing work on the development of psychological operationism mostly focuses on the contributions of Stevens (Feest, 2005; Hardcastle, 1995) and, outside Harvard, C. L. Hull's and E. C. Tolman's publications from the late 1930s and early 1940s (Feest, 2005; Green, 1992). In this paper, I aim to shed new light on the history of operationism by reconstructing the Harvard debate about the operational definition in the years *before* Stevens published his seminal articles. I argue that we can get a more complete understanding of Stevens' contributions if we better grasp the operationisms of his former teachers and direct colleagues at Harvard's Department of Philosophy and Psychology. Building on a large set of archival evidence from the Harvard University Archives, I argue that Stevens' colleagues mostly used operationism as an instrument to justify their existing perspectives on psychology and to attack approaches and theories from opposing schools.^2^

Thus far, the contributions of Stevens' direct colleagues have been somewhat understudied by historians of psychology. Skinner's views on operational definition have been thoroughly studied (e.g., Allen, 1980; Day, 1969; Flanagan, 1980; Moore, 1985) but these discussions focus almost exclusively on his *rejection* of certain variants of operationism after the mid‐1940s. Some scholars briefly discuss Boring's influence on Stevens in the year leading up to the publication of the latter's papers (e.g., Feest, 2005, pp. 138–140; Hardcastle, 1995, pp. 420–422), but little attention has been paid to the former's independent contributions to the development of psychological operationism. Walter (1990) and Grace (2001) do mention Boring's and McGregor's paper in their discussions of the *reception* of Stevens' operationism but they fail to notice that this paper antedates Stevens' articles. If we are to better understand Stevens' seminal publications, we need a more complete account of the contributions of his colleagues Skinner, Boring, and McGregor.

This paper is structured as follows. After summarizing the main features of Bridgman's operational approach (Section 2), I reconstruct the way in which Skinner used Bridgman's plea to rework the foundations of his “science of behavior” (Section 3). Next, I discuss the complex relationship between Skinner and Boring, showing how the two clashed about the merits of the behaviorist approach (Section 4). I analyze Boring's operationist turn between 1930 and 1934 (Sections 5 and 6) and reconstruct the ways in which he and his student McGregor used operationism to justify the scientific status of psychophysics (Sections 6 and 7). Finally, I turn to Stevens' development and take stock by comparing his views with those of his colleagues (Sections 8 and 9).

## 2 BRIDGMAN'S PLEA FOR CONCEPTUAL HYGIENE

Percy Williams Bridgman was a Harvard physicist who worked on the physics of high pressures and was the first person to create a device that could create pressures of more than 40.000 MPa. Next to his experimental work, which would be awarded the Nobel Prize in 1946, the Harvard professor was particularly interested in the philosophical foundations of physics, as he had been intrigued by the methodological and epistemological implications of the recent revolutions in relativity and quantum theory. In his book *The Logic of Modern Physics*, Bridgman (1927, pp. 9–10) attempted to create a way out of the conceptual confusion caused by the advances in modern physics, trying to formulate “something approaching […] to a systematic philoshy of all physics which shall cover the experimental domains already consolidated as well as those which are now making us so much trouble.”

When Bridgman published his *Logic* in 1927, he had no idea that his book was to have a tremendous impact on the course of experimental psychology. Nor did he foresee that his views would be turned into a proper “ism”—that scholars would use his book to proclaim that the “[o]perational doctrine makes explicit recognition of the fact that a concept […] has empirical meaning *only* if it stands for definite, concrete operations” (Stevens, 1935b, p. 517, my emphasis). For, Bridgman believed, he was not defending a strict methodological doctrine; he was merely defending an operational “attitude” or “approach.” Although he opens his book with a claim about the meaning that seems to entail a strict criterion of empirical significance (“In general, we mean by any concepts nothing more than a set of operations; the concept is synonymous with the corresponding set of operations,” p. 5), he also warns the reader that “[i]t will never be possible to give a clean‐cut logical analysis of the conceptual situation.” Our concepts, Bridgman (1927, p. 25) maintained, are simply too “hazy.” A year later, Bridgman (1928) repeated this warning in a review of Norman Campbell's *An Account of the Principles of Measurement and Calculation*—a book that seems to defend an equally rigorous view about meaning. In his review, Bridgman (1928, p. 999) mentioned that he is “not in sympathy with the […] view […] that there are in nature things which may be defined with the complete logical precision which we have come to associate with the entities of the mathematician.”

Rather than offering a strict criterion of empirical significance, Bridgman's book was mostly a plea for conceptual hygiene: if Einstein's revolution in physics has taught us anything, Bridgman argued, it is that the concepts of physics are inadequate.^3^ Einstein's revolt would not have been necessary if we had been more careful in our use of the concepts “time” and “simultaneity”—if we had adopted a more “critical attitude toward our whole conceptual structure” (1927, p. 1). For, Bridgman argued, we did not need any *experimental* discovery to find out what Einstein discovered; an operational analysis of the *concept* of “simultaneity” would have sufficed to at least leave open the possibility that simultaneity is not absolute, a conclusion which would have prevented Newton from building his physics on an unwarranted assumption. As a result, if we want to circumvent crises in the foundations of physics in the future, we have to be more careful—we have to submit the concepts of physics to operational analysis.

To explain what he means when he urges for an “operational analysis,” Bridgman extensively discusses the concept of “length” in the first chapter of *The Logic of Modern Physics*.^4^ If we thoroughly analyze the operations we use in measuring length, Bridgman argues, we have to conclude that even our use of this very basic concept rests on unwarranted assumptions: we use different operations to measure length in different circumstances without asking ourselves whether we can actually maintain that all these operations are measuring the same “property.” In ordinary situations, we use rods and rulers to measure length, whereas in astronomy, distance is measured by trigonometric triangulation, which is an entirely different operation:

We, thus, see that in the extension from terrestrial to great stellar distances the concept of length has changed completely in character. To say that a certain star is 10^5^ light years distant is actually and conceptually an entire[ly] different *kind* of thing from saying that a certain goal post is 100 meters  distant *(1927, pp. 17–18)*

If we cannot show that these different procedures yield similar results in similar circumstances, Bridgman concludes, we have to accept that we are dealing with different notions. We cannot just assume that “ruler length” and “triangular length” are the same concepts.

## 3 SKINNER'S PLAN

Bridgman's book quickly received a considerable amount of attention. *The Logic of Modern Physics* was selected as one of the “Forty Notable American Books of 1927” by the American Library Association (MacMillan to Bridgman, August 4, 1928, HUG 4234.12, Folder 1) and it was favorably reviewed in a host of journals, including general periodicals like *The Nation*, *The Observer*, and *The Saturday Review* (Verhaegh, 2020). In the academic world, especially psychologists were fairly quick to see the potential of Bridgman's approach, perhaps because they were inspired by his remark that “[i]t would doubtless conduce greatly to clarity of thought if the operational mode of thinking were adopted in *all* fields of inquiry” (1927, 30, my emphasis). Indeed, in one of the first discussions of Bridgman's book in the psychological literature, H. M. Johnson argues that a “rigorous application of [Bridgman's] method to the analysis of psychological ‘problems' would show a huge proportion to be spurious, and would forestall much unnecessary and irrelevant experimentation” (1930, 113n). Johnson, a psychologist from Pittsburgh, illustrated his argument by applying Bridgman's approach to one of the most fundamental concepts in psychophysics. Using the tools that Bridgman had offered in his *Logic*, Johnson objected to the widespread assumption that Fechner's law should be interpreted as expressing the relation between physical stimuli and the intensity of our sensations. If we express Fechner's law as

(1) γ=k⁎log(β/b)

in which β is the size of the stimulus, b is the threshold value, and k is a logarithmic constant, Johnson argued, we should not interpret γ as expressing “intensity of sensation.” Rather we should equate γ with the *physical operations* which we use to determine k, b, and β. If we want to argue that γ is something more and can be equated to the intensity of introspectional sensation (*I*), we need to specify an *independent* operation which we can use to determine *I* and examine whether *I* is equivalent to γ:

[The meaning of γ] is the operations that create it. It is often discussed, however, as if it were interchangeable with another concept, which we may call the *intensity of introspectional sensation*, and denote by the symbol *I*. The question is, whether *I* = γ. To give an answer that satisfies the operational criterion, we must first specify the operations by which *I* is determined […] if no feasible operations can be specified […] the question whether *I* is equivalent to γ becomes a question whether an undefined concept is equivalent to a defined concept; […] Busy people usually do not entertain such a question very long after they have detected its character *(Johnson*, 1930, *p. 116)*

Bridgman's criterion, in other words, allowed Johnson to do what behaviorists had been doing for almost 20 years, to criticize the appeal to introspection on methodological grounds. This time around, however, Johnson could appeal to methodological prescriptions developed by a world‐renowned physicist and formulate the even stronger objection that the notion of introspectional sensation is *meaningless* if it cannot be operationally defined.

Some behaviorists soon started to see the appeal of the operational approach as well. Among them was B. F. Skinner, at the time still a Harvard graduate student. Skinner (1979, p. 11), who had been “converted to the behavioristic position” after reading Watson's *Behaviorism*, started his graduate program in 1928, 3 years before Stevens. It did not take long, however, before Skinner () discovered that his teachers at Harvard were not particularly interested in behavioristic approaches to psychology and he quickly turned to W. J. Crozier and Hudson Hoagland at the Department of Biology, since they *were* discussing Pavlov and conditioned reflexes in their physiology courses (1979, pp. 13–15, 21). Influenced by the two, Skinner started to specialize in what he himself called “the behavior of intact organisms” (1931b, 427), a field of study which aims to analyze behavior without delving into the underlying physiological mechanisms.^5^ Despite his very limited background in the study of behavior, Skinner soon published an experimental study on what he called “eating reflexes” in rats. In short, Skinner showed that the rate at which deprived rats eat their daily rations of food over the course of two hours is lawful and can be mathematically described by the power function

(2) $$N=Kt^{n},$$

where *N* = the amount of food eaten at time *t* and *K* and *n* are constants. Even more surprisingly, Skinner (1930, p. 437) showed that *n* is an approximately constant magnitude (between 0.67 and 0.71), even when the conditions of the experiments (food size, rat, and exposure to food on the day before the experiment) varied. In a letter to his parents, Skinner cheerfully reported:

I got […] some remarkable results from the data of my experiment. […] In a word, I have demonstrated that the rate in which a rat eats food, over a period of two hours, is a square function of the time. In other words, what heretofore was supposed to be “free” behavior on the part of the rat is now shown to be just as much subject to natural laws as, for example, the rate of his pulse *(Skinner*, 1979, *p. 59)*

It was about the same time that Skinner learned about *The Logic of Modern Physics* from Cuthbert Daniel, a former MIT student in chemical engineering who had come to Harvard to work with Bridgman (ibid., p. 41).

Bridgman's approach chimed in with Skinner's perspective on the philosophy of science, which was also shaped by two books he read for L. J. Henderson's and George Sarton's courses on the history of science: Ernst Mach's *The Science of Mechanics* and Henri Poincaré's *Science et Méthode*.^6^ Inspired by these positivist readings, Skinner decided to study the history of the *concept* of “reflex,” which had been fundamental in his experimental study on eating reflexes. Starting with Descartes' account in *Traité de l'homme*, Skinner (1931b) reconstructed the history of the notion in both psychology and physiology and published it as “The Concept of the Reflex in the Description of Behavior.” In this paper, Skinner (1931b, p. 450) concluded that “[a] reflex […] has no scientific meaning apart from its definition in terms of […] experimental operations”, an insight he directly attributed to Mach, Poincaré, and Bridgman:

The reader will recognize a method of criticism first formulated with respect to scientific concepts by Ernst Mach and perhaps better stated by Henri Poincaré. To the works of these men and to Bridgman's excellent application of the method to more modern concepts the reader is referred for any discussion of the method *qua* method *(1931a, p. 427)*

In the remainder of his paper, which also served as the first part of his dissertation (Skinner, 1931a), the young graduate student proposed a definition of “reflex” in terms of publicly observable operations. In short, Skinner uncovered a definition that satisfied what he perceived to be Bridgman's operationist's strictures. Where psychologists and physiologists had often appealed to the “involuntary” or “unconscious” character of reflexes in their definitions—concepts that, Skinner (1931b, p. 445) believed, are problematic from an operationist point of view—Skinner argued that a reflex is nothing more than “an observed correlation of two events, a stimulus and a response.” For *correlations* between stimuli and responses *are* directly observable.

Skinner's paper on the notion of “reflex” set into motion a new program of defining the core concepts of psychology in operationist terms; within a year after his reflex paper, Skinner (1932a, 1932b) also published two articles on the concept of “drive.” Indeed, in a note titled “Plan of the campaign for the years *30*‐*60*,” Skinner listed “operational definitions of all psychological concepts” as one of his two main career goals for the next decades:

Plan of the campaign for the years 30–60

1. Experimental description of behavior. Continue along the present lines. Properties of conditioning, extinction, drives, emotions, and so forth. No surrender to the physiology of the central nervous system. […]

2. Behaviorism versus Psychology. Support behavioristic methodology throughout. Operational definitions of all psychological concepts^7^ (November 17, 1932, HUGFP 60.50, Box 3, Folder 6).

Skinner, in other words, aimed to use Bridgman's operational approach to improve the foundations of behaviorism. Yet, Skinner did not only use operationism for constructive purposes. In his unpublished 1930s notebook *Sketch for an Epistemology*, which contains his notes for a monograph he started writing in the early 1930s, Skinner also used his newfound methodological principles to criticize alternative theories about mind and knowledge, including some of the very doctrines that had once been defended by Mach. One of Skinner's reasons for writing the book on epistemology, these notes show, was his dissatisfaction with the widespread influence of *phenomenalism*—the radical empiricist view that physical objects are nothing but constructions out of primary sense experiences (“phenomena”). In the early 1930s, phenomenalism was a popular view about the nature of our knowledge about the physical world, defended by epistemologists, psychologists, and influential physicists like Arthur Eddington and James Jeans. When Skinner started working on his *Sketch*, both Eddington (*The Nature of the Physical World*) and Jeans (*The Mysterious Universe*) had just published books that relied on strongly phenomenalist conceptions of science. Skinner, on the other hand, strongly objected to the growing popularity of phenomenalism. In one his notes in *Sketch for an Epistemology*, he described the situation as follows:

Recent trends are toward a solution of the dilemmas of physics in terms of a theory of knowledge. It would be a pity if physicists in turning to epistemology should take up an out‐moded scheme of mind, which presents as many difficulties in its own systematization as the physicist is trying to rid himself of in physics. Jeans and Eddington are already out of the frying pan into the fire. This movement cannot be traced to one source. On the one hand lies positivism, on the other Ernst Mach *(HUGFP 60.50, box 3, folder 5, my transcription)*

Skinner's reference to Mach seems surprising considering the fact that his work on the notion of “reflex” was modeled on the latter's *The Science of Mechanics*. Still, Skinner's early notes show that he strongly disagreed with Mach's *The Analysis of Sensations*, a book he read as a staunch defense of phenomenalism. According to Skinner, Mach had it exactly backward: we do not need a phenomenalistic analysis of science, we need a scientific analysis of “phenomena”:

Mach reduces the concepts of science to a subjective basis […] we can return to an objective expression by asking him for a definition of sensation. This can only be supplied […] in terms of Mach's behavior (as a scientist). Thus while Mach makes science personal (and therefore private), the definition of sensation makes it again public, i.e. a matter of human behavior *(HUGFP 60.50, box 3, folder 5, my transcription)*

Whereas epistemologists and mentalistic psychologists aimed to secure our scientific knowledge by reconstructing our fallible concepts and theories out of “indubitable” sense experiences, Skinner aimed to revert the picture: we should not aim to ground science in sensation, we should ground sensation in behavioral science.

It is no coincidence that Skinner demands a public *definition* of sensation in arguing against phenomenalism. *Sketch for an Epistemology* is written from what Skinner considered to be an operationist perspective. Skinner notes that we ought to try “the operational method on ‘knowledge,'” that knowledge should be “operationally defined,” and that historical advances in the sciences might be explained by the fact that scientists (unconsciously) relied on “a positivistic (Machian, Poincarean, Bridgmanian) philosophy of science” (HUGFP 60.50, Box 3, Folder 5). Skinner, in other words, argued against the phenomenalists' attempts to reduce science to sense experience by applying Bridgman's call for conceptual hygiene to notions like “knowledge,” “mind,” and “sensation”; the phenomenalists' proposals are simply meaningless if they do not provide an operational definition of “sense experience.” Skinner, of course, believed that the only viable definition of sense experience would be a behavioristic one.

## 4 SKINNER VERSUS BORING

Skinner's dissertation on the notion of reflex was supervised by E. G. Boring, the director of Harvard's psychological laboratory. Unlike Skinner, Boring (1929) was far from a behaviorist at the time as he had criticized the movement in the first edition of his seminal *A History of Experimental Psychology*. According to Boring, behaviorists face a dilemma: either they incorporate the problems of traditional psychology (e.g., questions about consciousness, introspection, and sensation) and translate its concepts into behaviorist terms—in which case their hypotheses become as imprecise as the theories they are supposed to replace—or they decide to ignore these traditional objects of study and their theories become strictly physiological:

Behaviorism […] is very closely allied to physiology. Probably the only way it is to be distinguished from physiology is by its problems, which came, not from within itself, but from the conventional psychology […] As behaviorism began to absorb most of the content of the older psychology […] much of the original precision of physiological method was lost^8^ *(1929, pp. 585–586)*

Behaviorism, in other words, either reduces to physiology (in which case it does not compete with conventional psychology) or incorporates the questions of traditional psychology, thereby importing the very problems it was supposed to dissolve.

Skinner was annoyed by passages like these as he was convinced that there *is* a middle way between physiology and conventional psychology. Indeed, Skinner's operational definition of the concept of reflex implicitly aimed to show that we can avoid both horns of Boring's dilemma: if we operationally define a reflex as a *correlation* between stimulus and response, Skinner argued, we do not have to worry about intervening physiological variables (i.e., events between stimulus and response) nor do we have to incorporate the imprecise concepts that were associated with reflexes in traditional psychology (e.g., “involuntary behavior” or “unconscious behavior”).

Skinner's first major experimental study on eating reflexes in rats (Section 3) also illustrates his idea that there is a middle way between physiology and traditional psychology. In the paper, we have seen, Skinner shows that the rate at which rats eat food over the course of 2 hours can be described by a power function. We can predict eating behavior without using the imprecise concepts of conventional psychology (e.g., “hunger” or “motivation”) and without studying the rat's physiology; it is enough to functionally describe the relation between stimulus and response.^9^

When Boring (1932) repeated his argument against behaviorism in an article in *Science* (“It is worth noting that behaviorism owes its *ism* to consciousness. And what would it be without its *ism*? Well, it would be physiology,” p. 33), Skinner responded furiously. In a letter to his friend (and former fellow graduate student) Fred Keller, Skinner writes:

Have you seen Boring's article in *Science*? It's scandalous. Probably the most astonishing misunderstanding of behaviorism yet attained, even by Boring. “Without consciousness, behaviorism has nothing left but the nervous system.” That sort of thing. Well, here's *to* him, the stupid son‐of‐a‐bitch.^10^ *(ca. 1932, HUA accession 14328, Box 1, Folder 4)*

The direct source of Skinner's opposition to Boring, however, was not the latter's argument against behaviorism. The seeds of Skinner's aversion had already been sown in October 1930, when Boring heavily criticized the first version of his dissertation. In a five‐page letter, Boring dismissed the first part of Skinner's thesis (on the notion of the reflex) in extraordinarily strong words. According to Boring, the central structure of Skinner's thesis was utterly confused—there is no use in giving the notion of reflex a new meaning:

You are making an argument for keeping the word *reflex* and giving it a new, broader and relatively strange meaning. […] Now this is a pretty bold proposal and I question its value. You have given a very broad, strange, almost bizarre meaning to the word *reflex*. You have taken it away from the constrained anatomical reflex‐arc meaning, and you have equated it to the concept of the psychological fact‐as‐relational‐correlation which already has terms for itself. What is the use?

[…]

I do not mean to be harsh, but your very versatility and you[r] polemical c[le]verness make it necessary for older people to tell you bluntly where they think the trouble lies. Otherwise you might go on through life doing half‐baked work which wins applause from the uncritical and the unsophisticated […] and thus never realizing that your work was superficial^11^ *(Boring to Skinner, Oct. 13, 1930, HUGFP 60.7, Box 1, Folder 2)*

Clearly, Boring failed to see the relevance of Skinner's attempt to provide an *operational* definition of “reflex.” Although some of Boring's arguments seem justified, he appears to have misunderstood the purpose of Skinner's project. To some extent, Boring's misinterpretation of Skinner's aims is understandable, however. Even in the published version of his paper on the notion of reflex, Skinner does not really discuss his methodology except for the very brief reference to Mach, Poincaré, and Bridgman quoted in Section 3 and it is unclear whether this passage was already included in the version Boring read.^12^

Skinner seems to have been aware that Boring simply failed to understand the nature of his project. In the margins of Boring's second letter with comments on his thesis (December 9, 1930), Skinner writes: “I'm not trying to do all you think” and “I want to define a science of the descrptn. of behavior […] and to define the concept of the reflex so that it is workable” (Skinner's marginalia, HUGFP 60.7, Box 1, Folder 2, my transcription).^13^ Yet, instead of explaining this methodological background and writing “the introductory paragraph” that Boring asked for, Skinner chose to resubmit his thesis essentially unchanged, informing Boring that the first draft had “been read by four other responsible people” and that “they have all expressed, in almost the same words, the opinion that it is a good job which needed to be done and has been done well.” Referring to the last part of Boring's above‐quoted comments, Skinner adds:

What am I am to think? Am I to assume that these four are uncritical and unsophisticated, and that if my work has won applause, it has nevertheless been superficial? Must I admit that I am “too clever always to be thorough?” *(Skinner to Boring, December 14, 1930, HUG 4229.5, Box 53, Item 1180)*

Instead of resolving the misunderstanding by clarifying the nature of his project, in other words, Skinner chose to dig in his heels. He informed Boring that the final decision “will be up to the committee” and ended his letter with a couplet from Thomas Hood's “Bridge of Sighs”: “Owning her weakness, her evil behavior,/and leaving with meekness her sins to her Saviour” (ibid.).

## 5 BORING'S PROTO‐OPERATIONISM

Boring knew that his feedback on Skinner's dissertation had been extraordinarily critical. A letter to Herbert Langfeld, however, shows that there were two sides to Boring's assessment: although he genuinely believed that his student's work was “epistemologically naïve,” he also saw that Skinner's *experimental* work was unusually innovative. Ten months after Skinner obtained his Ph.D., Boring summarized the events as follows:

I was not on the thesis committee, since I had criticized Skinner's views so vigorously in advance that I thought that he would feel that the committee had been stacked against him if I were to be on it. […] The point i[s] […] that Skinner, who thinks of himself as a genius, was dealing with a fundamental issue of methodology where he was quite inexpert […] On the other hand, believe it or not, Skinner is an excellent experimentalist and has done some of the cleverest apparatus work with animals (self‐recording devices) that we have had done here *(Boring to Langfeld, November 4, 1931, HUG 4229.5, Box 33, Item 735)*

Boring's acknowledgment of Skinner's experimental talents in the above letter shows that the theoretical misunderstanding between the two is unfortunate. For there is quite some evidence that Boring was actually sympathetic to operationist thinking—that is, there is quite some evidence that Boring would have been more sympathetic to his student's epistemological project if the latter had explained the nature of his project in more detail.^14^

First of all, Boring's criticism is primarily methodological. He complains that there is no reason to redefine the notion of reflex considering that the “concept of the psychological fact‐as‐relational‐correlation […] already has terms for itself.” Boring's arguments do not entail that he would have rejected Skinner's *definition* if he *had* understood the latter's purposes. On the contrary, Boring makes clear that he is “very sympathetic” to Skinner's definition:

The reflex is a fact, and therefore a relation, statable in terms of an experimentally observed correlation. You would have to know, even if only from dropping into Psychol.31 this fall, that this line of thought is one with which I am personally very sympathetic *(Boring to Skinner, October 13, 1930, HUGFP 60.7, Box 1, Folder 2)*

This remark does not stand on its own. Throughout the 1920s and early 1930s, Boring often defended proto‐operationist views that reveal a strong preference for strictly empirical definitions.^15^ In debates about the question of whether intelligence tests measure what they ought to measure, for instance, Boring (1923, p. 35) was well known for the view that “intelligence as a measurable capacity must at the start be defined as the capacity to do well in an intelligence test.” Instead of viewing “intelligence” as some hidden, mysterious property that escapes precise definition and can only be measured by approximation, Boring equated intelligence with the operations we use to measure it. Years before Bridgman published *The Logic of Modern Physics*, in other words, Boring was already arguing that intelligence *is* “what the tests test” (ibid.).^16^

Second, when Boring (1933), in *The Physical Dimensions of Consciousness*, started to defend a mind/brain identity theory—arguing that mental processes *are* physical processes—he primarily used an operationist argument to justify his claim. Relying on experimental work that shows that there are strong correlations between mental and neurological processes, Boring argued:

If we were to find a perfect correlation between sensation *A* and neural process *a*, a precise correlation which we had reason to believe never failed, we should then identify *A* and *a*. If introspection yielded *A*, it would yield knowledge of the nervous system; and, conversely, the physiologist would, in knowing about *a*, know about sensation *(Boring, 1933, p. 14)*

Instead of presupposing that mental processes and neural processes are *intrinsically* different, and, therefore, unidentifiable kinds of events, Boring implicitly relied on the operationist assumption that the meaning of a concept is fully determined by its observable effects and, hence, that “a perfect correlation *is* identity” (ibid., 16, my emphasis).^17^

Boring's theoretical orientation is a decidedly different one than Skinner's. Boring never abandoned talk about “mental” processes, he never adopted a behaviorist conceptual framework, and he never had any methodological concerns about physiological data. Still, the above arguments show that the two shared an (in Boring's case, still implicit) penchant for operationist definitions; both Skinner and Boring strongly felt that the psychologist's concepts ought to be defined in terms of publicly observable behavior. It is, therefore, doubly ironic that their theoretical dispute in the early 1930s turned precisely on Boring's misunderstanding of the former's operationist project.

So how might we explain Boring's and Skinner's shared operationist leanings, considering their strong disagreements about behaviorism, introspection, and physiology? It is my contention that the answer can be found in a paper Boring wrote in the same month in which he “vigorously criticized” Skinner's thesis. In the paper—titled “The Psychologist's Circle”—Boring argues that the most fundamental distinction in psychology is the opposition between psychologists who believe that all scientific facts are inferred from sense experience (he mentions Wundt and Köhler as examples) and psychologists who believe that “the phenomenal processes of introspective psychology must be regarded as dependent upon the nervous system” (e.g., Avenarius and Külpe). The first perspective entails that psychology is propaedeutic to physics whereas the second perspective implies that physics offers the most fundamental perspective (Boring, 1931, pp. 178–179).

Skinner, we have seen, attempts to dissolve this circle by choosing something like the latter option. Although he certainly does not aim to study *physiological* processes, he strongly rejects the Wundtian (phenomenalist) horn of Boring's dilemma (see Section 3). Now, despite all their differences, Boring makes a similar point. Although he does not use a behaviorist vocabulary, Boring implicitly defends a variant of operationism in arguing that we can dissolve the psychologist's circle if we recognize that *both* sides of the divide are essentially working with *constructed* concepts—that is, if we acknowledge that physical *and* psychological facts are “mediate.” According to Boring (1931, pp. 180–181), the “experience‐first” psychologists are simply “wrong in implying […] that the subject‐matter of psychology is in any way more “phenomenal,” “immediate,” or “direct” than the subject matter of physics.” The physicist and the psychologist, in other words, are in the same boat; since there “is no way of getting at 'direct experience,'” both the concepts of physics *and* the concepts of psychology are “systematic constructs” (1933, p. 6).

Of course, as soon as one recognizes that the concepts of psychology are theoretical constructs, the question arises *how* they are constructed—that is, how they are related to our experimental data. As soon as he acknowledged that the concepts of psychology are precise that—concepts—Boring faced the very question Bridgman had asked about physical concepts: how can we ensure that psychologists are talking about the same things when they are discussing their theories about “experience,” “consciousness,” and “sensation”?

## 6 McGREGOR'S THEORY OF MEASUREMENT

Considering his novel views about psychological concepts in the early 1930s, it is perhaps no surprise that Boring became heavily interested in Bridgman's operational approach. Indeed, he extensively discussed Bridgman's *The Logic of Modern Physics* in his 1934 graduate seminar on “The Data of Psychology,” a class that was attended by Douglas McGregor and Stanley S. Stevens. On the very last day of the seminar, Boring wrote Bridgman a note, informing him that he and his students had “been very much interested in the epistemology of operationalism” that winter (May 28, 1934, HUG 4229.5, Box 5, Item 106).^18^

When McGregor attended Boring's seminar, he was a second‐year graduate student. He had obtained a B.A. from Wayne University and had been admitted to Harvard Graduate School in 1932. Once in Cambridge, he had quickly shown an interest in the psychophysical projects that Boring was working on with Stevens (who had been admitted to Harvard a year before McGregor). After some brief excursions into experiments with mice and work on motor learning at Worcester State Hospital, McGregor wrote Boring about his wish to work on one of Boring's topics (“I can think of nothing I would like better than to be doing a problem under you,” July 17, 1933, HUG 4229.5, Box 38, item 812). This work on Boring's projects eventually resulted in a dissertation about color sensitivity (defended in June 1935)—a thesis which, in Boring's opinion, “definitively alter[ed] the prevailing conceptions about the nature of color blindness” (Boring to Tracy McGregor, April 12, 1935, HUG 4229.5, Box 38, item 813). A year before he defended his dissertation, McGregor participated in Boring's seminar and wrote “Scientific Measurement and Psychology,” the purpose of which is to show that recent “findings” about measurement in the natural sciences also have consequences for measurement in psychology:

recent concern about the validity of scientific measurement has aroused but slight interest among psychologists, possibly because they think that a great gulf exists between physical and psychological methodologies. It is the purpose of this paper to demonstrate that such a gulf does not exist […] If we, as psychologists, are to have measurement of any value whatever we must accept the logical restrictions imposed within other sciences *(1935, p. 246)*

Not surprisingly, the “modern theories of measurement” McGregor refers to are “operationist” theories, which claim that “an entity is adequately defined only in terms of the specific operations involved in its observation” (ibid., 247).

“Scientific Measurement and Psychology” is a significant contribution to the development of operationism because it modifies the discussion about the operational definition in two ways. First, it provides a novel answer to the question of what an operation ultimately is. Whereas Bridgman and Skinner, at least in their publications before 1935, do not attempt to *define* the term “operation,” McGregor offers something like an operationalization of the term “operation” as well: he suggests that operations are ultimately *discriminations*. No matter how complex our concepts are, if they are operationally defined, they can ultimately be reduced to a series of discriminations:

An object is said to be 10 centimeters long when, under specified conditions, the observer can detect no difference in length between the object and a portion of the scale comprising 10 centimeters […] With weight, a fundamental magnitude, the discrimination involves the pointer and the scale of a balance […] Even with “robot” measurement, such as that provided by the photo‐electric cell, calibration is necessary, and calibration involves discrimination *(1935, p. 248, 258)*

As a result, McGregor's claim that a concept is “adequately defined only in terms of the specific operations involved in its observation” can be restated as the claim that a concept is adequately defined only if we are physically able to discriminate between situations in which the concept does and does not apply.

A second way in which McGregor changes the discussion about operational definition is that he essentially turns operationism into a methodology for psychophysics. Although he opens his paper with the claim that he will be discussing the “importance of modern theories of measurement for *psychology*,” McGregor's paper is exclusively concerned with psychophysical research: his examples of psychological variables include only characteristically psychophysical variables like “[c]hromatic saturation, loudness, weight, pressure, sweetness, pain,” and McGregor and Stevens' own objects of study “visual brilliance […] tonal volume and tonal density” (ibid., 259–260).^19^ Whereas Skinner had primarily used operationism to justify his behaviorist approach to psychology, in other words, McGregor constructed an operationist theory of measurement to justify the approach Boring, Stevens, and he himself were using.

McGregor's two “innovations”—a discrimination‐based interpretation of operational definition and the application of operationism to psychophysical research—are intimately connected. For it is no coincidence that McGregor also views discriminational analysis as the most important instrument in the psychophysicist's toolbox: to study color sensitivity or tonal density *is* to study the discriminatory capacities of test subjects. In McGregor's paper, psychophysical measurement is reinterpreted as the measurement of the discriminatory capacities of a physical system: just as we can test the discriminatory capabilities of a digital kitchen scale by gradually increasing the amount of flour on its surface, we can examine a person's color sensitivity by examining when a subject is and is not able to discriminate between two colors.^20^

In (1) classifying operational definition as the guiding principle of measurement in the natural sciences, in (2) suggesting that operations are ultimately discriminations, and in (3) reinterpreting psychophysics as the study of discriminatory capacities, therefore, McGregor effectively turned psychophysics into natural science. Indeed, McGregor's concluding paragraph leaves no doubt that this was part of his agenda:

Psychological measurement, understood in operational terms […] *is* physical measurement. It always has been. And the psychologist, now aware that he is using no mysteriously unique scientific instrument (the observer), can, secure in his new self‐knowledge, proceed with his measurements, unimpeded by the hampering difficulties of the Cartesian dichotomy between mind and body. *(1935, pp. 265–266)*

When observers are reinterpreted as physical systems with certain discriminatory capabilities, in other words, the operationist circumvents questions about the nature of observing subjects and turns psychology (or better: psychophysics) into natural science.

## 7 BORING'S INFLUENCE

Boring wholeheartedly agreed with McGregor's conclusions. In a letter to Herbert Langfeld, he describes McGregor as one of his “up‐and‐comings” and praises the paper in extraordinarily strong terms:

I cannot escape from the belief that [McGregor's] paper says the most important thing about mental measurement that has been said in the last twenty or thirty years […]. Secondarily, in doing that and in appealing to operationism, he makes a secondary point in favor of monism and against a dualism, a point that seems to me so inevitable and convincing as to have me hopping up and down *(October 29, 1934, HUG 4229.5, Box 34, Item 736)*

Considering his proto‐operationist views in the 1920s and early 1930s (Section 5), it is no coincidence that Boring agreed with McGregor's conclusions. In addition, he had examined a very similar position in his 1921 paper “The Stimulus‐Error.” In response to the so‐called quantity‐objection to psychophysics—the problem that it is impossible to find quantitative correlations between physical stimuli and sensations because sensations do not admit of quantitative description—Boring (1921, p. 459) discussed a solution he dubbed the “psychology of capacity,” which is the view that psychophysics is not measuring *sensation* but “capacity‐for‐discrimination.” Rather than viewing psychophysics as correlating mental and physical phenomena, the “psychology of capacity” approach claims that psychophysics only describes physical stimuli and “errors of observation incurred in observing the stimuli” (ibid.).^21^

The similarities between Boring (1921) and McGregor (1935) raise the question to what extent Boring influenced his student. Luckily, there is some archival evidence that sheds light on the way in which McGregor's paper was produced. In the above‐discussed letter to Langfeld, for example, Boring admits that he played a substantial role in writing the paper and that he feels as if the paper is partly his:

It is not a joint publication by McGregor and me, because he has really done all the hard work. On the other hand I have spent three strenuous seven‐hour Sundays on it and innumerable other little conferences, so that many of the sentences I know by heart, and I feel as if the paper belonged in part to me *(October 29, 1934, HUG 4229.5, Box 34, Item 736)*

In a letter to Stevens, moreover, Boring claims that McGregor's first draft was not very good and suggests that he has read and criticized six drafts before it was ready for publication (ca. November 1934, HUGFP 2.10, Box 1, Folder 3). It is likely, therefore, that McGregor's views were heavily influenced by Boring, especially because he was still a second‐year graduate student when he wrote the paper. Boring refused to accept a coauthorship; however, because he was strongly opposed to the practice of co‐authorships between students and supervisors.^22^ Boring only agreed to write a signed footnote on the opening page of McGregor's (1935, 245n1) paper—a footnote in which he states that “McGregor […] and I have worked in close collaboration” and that he is “in hearty accord with everything [McGregor] says.”

## 8 STEVENS' MANIFESTO

McGregor's paper was not the only iron in Boring's fire. For, in the fall semester of 1934, Stanley S. Stevens was working on an operationist paper as well—a paper, moreover, that also (1) primarily addresses psychophysical research and (2) defends the position that “*[d]iscrimination* is the *sine qua non* of any and every operation” (Stevens, 1935a, p. 324, original emphasis). Stevens, as has been well documented in the literature on the development of operationism, also argued that operations are ultimately discriminations and that it is the goal of psychology to “measure the discriminatory capacity of the organism” (ibid., 325). The similarity between McGregor's and Stevens' papers is especially striking in their discussions of the status of psychological research. Like McGregor, Stevens argues that “the relation of the psychologist to the object of his investigation is fundamentally not different from that of any other scientist to his subject‐matter”:

The body of psychological science as it now stands relates to verifiable responses obtained from organisms treated as objects of study by capable experimenters […] The same relationship must obtain in all scientific psychology. The utility of this type of “objective” approach lies in the fact that all operations involved are essentially public and repeatable. A human being enters the situation as a complex physical system whose characteristics can be investigated by a method essentially the same as the methods used for the investigation of all physical systems^23^ *(ibid., 328)*

Stevens wrote “The Operational Basis of Psychology” during his first year as a National Research Council Fellow at Harvard Medical School, where he was working with Hallowell Davis. Before his fellowship, however, Stevens had been Boring's student and assistant for 3 years; and it was in this capacity that he first encountered psychophysical research. In April 1932, only 2 months after he had decided to pursue a degree in experimental psychology (Stevens, 1974, p. 18), Boring had asked Stevens to read the first draft of *The Physical Dimensions of Consciousness* to obtain some general comments from a non‐specialized reader.

One can imagine the impact this advisory role had on Stevens' development, especially because it was his first encounter with serious psychophysical research.^24^ And indeed, when the book came out in January 1933, Stevens proudly reported his contribution in his notebook:

Prof. Boring's book on the physical nature of consciousness will be out tomorrow […] He refer[r]ed to it in conversation as *our* book. I acknowledged the compliment. Reading the manuscript was a particularly pleasant task—a privilege rather than a job; and especially so since Prof. Boring's impatience with the dualism which he nominates for the ash heap is very similar to my own *(January 22, 1933, HUGFP 2.45, Box 1, Folder 1)*

In that same semester, Stevens decided to write his thesis in psychophysics and informed Boring that the subject of his dissertation would be “the measurement of intensity and volume in audition” (Stevens to Boring, October 14, 1932, HUG 4229.5, Box 54, Item 1215), a relation Boring (1933, p. 82) had classified as “somewhat of a puzzle” in *The Physical Dimensions of Consciousness*. Not even 2 years later, Stevens (1935a, p. 325), would be writing the seminal paper in which he argued that it “is the sole business of psychology to test and measure the discriminatory capacities of the organism.”

Gary Hardcastle has argued that Boring played only a limited role in the development of Stevens' operationism.^25^ Considering (1) the background of the relation between Boring and Stevens, (2) Boring's operationist development in the 1920s and early 1930s, (3) Boring's significant influence on McGregor's paper, and (4) the similarities between McGregor's and Stevens' papers; however, I think that his account needs to be reevaluated. Although it is difficult to determine the exact dynamic of the relation between Boring, Stevens, and McGregor during the seminar meetings, it is likely that Boring played a substantial role in shaping both Stevens' and McGregor's views as he was both their supervisor and had been gradually evolving toward a very similar position for more than 15 years.

There is some additional evidence for my thesis in Stevens' own recollection of the early 1930s. For Stevens himself has explicitly argued that Boring had already come close to formulating an operationist perspective in *The Physical Dimensions of Consciousness*:

*The Physical Dimensions of Consciousness* […] showed how far Boring in 1932 had departed from the Titchenerian tradition, it was […] an effort (as part of Boring's enduring effort) to achieve clarity for the meaning of the basic terms of psychology, terms like consciousness, sensation, and the rest […] Boring knew whereof he wanted to escape, but at that stage he was too entangled in his past to effect a clean restatement […] it now seems clear that an operational restatement of psychology's basic concepts was Boring's real target. The [1935 paper on operationism] appeared under my name, but it can be proved from page upon page of editorial criticism that large segments of those papers were generated more by Boring than by me *(Stevens*, 1968, *pp. 596–597)*

Indeed, Boring's 12 pages of typed, single‐spaced comments on the four drafts of “The Operational Basis of Psychology” strongly suggest that Boring still had a major influence on Stevens' development after he started working at Davis' lab in September 1934. For although Hardcastle (1995, p. 420) is correct in concluding that about two‐thirds of Boring's comments are “concerned *solely* with style,” the comments also show that Boring and Stevens were still regularly discussing substantial issues in person.^26^

More importantly, Boring's extensive comments on Stevens' drafts reveal that Boring was the driving force behind the manifesto‐like language of Stevens' papers. Boring's comments show that the strongest sentences of the paper (e.g., “the revolution to end the possibility of revolutions” and “it is the sole business of psychology to test and measure the discriminatory capacities of the organism”) are Boring's (HUGFP 2.10, Box 1, Folder 3). In general, it appears that Boring liked the idea of establishing a psychological school of his own. For it was Boring who cheerfully reported to Stevens that “operationism is getting identified in psychology with you and me and Harvard” when people started to ask him questions about operationism (October 12, 1935, HUGFP 2.10, Box 1, Folder 3), it was Boring who organized the influential 1945 symposium on operationism (Langfeld to Stevens, August 3, 1944, HUGFP 2.12, Box 2, Item 16), and it was Boring (1946), who would identify operationism as one of the eight main schools in psychology in an encyclopedia article. In this entry, Boring even goes as far as classifying behaviorism as a variety of operationism (“operationism […] includes behaviorism”) and concludes that, as a result, “the modern dichotomy in systematic psychology lies between operationism […] and phenomenology” (ca. June 1946, HUG 4229.5, Box 6, Item 113).^27^

## 9 CONCLUSION

In this paper, I have reconstructed some of the debates about operationism at Harvard's Department of Philosophy and Psychology in the late 1920s and early 1930s to get a more complete understanding of the context in which Stevens published his call for a “revolution that will put an end to the possibility of revolutions” in psychology. I have discussed the operationist approaches developed by (1) B. F. Skinner, who adopted an explicitly operationist method in both his dissertation and his unpublished notebook *Sketch for an Epistemology*; by (2) Douglas McGregor, who applied Bridgman's perspective in his paper “Scientific Measurement and Psychology”; and by (3) E. G. Boring, who played an important role in supervising McGregor and Stevens and defended proto‐operationist views throughout the 1920s and 1930s.

I have argued that these Harvard psychologists mostly used Bridgman's call for conceptual hygiene to justify their existing approaches to psychology and to attack theories and concepts from opposing schools. Skinner, we have seen, used Bridgman's methodological strictures both to argue against “mentalistic” and “phenomenalistic” concepts and to create space for his “science of behavior,” a discipline that would circumvent Boring's dilemma that behaviorism either reduces to physiology or incorporates the problems of traditional psychology. Boring failed to see the merits of Skinner's operationist approach, repeatedly classifying him as ‘epistemologically naïve', but he quickly adopted a variant of operationism once he realized that Bridgman's perspective fitted with his views about psychological concepts as outlined in *The Physical Dimensions of Consciousness*. Two of his students—McGregor and Stevens—in turn, used operationism to validate Boring's psychophysical research. This is especially clear in McGregor's paper on scientific measurement in psychology, which used Bridgman's perspective to argue that psychophysics should be viewed as natural science.

In the end, it was Stevens' two‐part paper that put psychological operationism on the academic map. In these papers, Stevens presented operationism as a “straightforward procedure for the definition and validation of concepts” (Stevens, 1935a, p. 323) but in practice, the procedure turned out to be much less clear‐cut than he presumed. Indeed, when Skinner read Stevens' papers—especially the passage in which the latter claimed that “[i]t is the sole business of psychology to test and measure the discriminatory capacities of the organism” (ibid., p. 325)—he responded by dismissing the latter's psychophysicist's variant of operationism:

That is your heritage from Wundt and Fechner […] What is happening in a discrimination, and what properties organisms actually *do* use in setting up classes (concepts, objects) are far more important questions. […] As I have said so many times that I blush to say it again—if you approach the behavior of an organism as an object of scientific study and set to work, it would be a long time before you would reach the field of discriminatory capacity *(June 16, 1935, HUGFP 2.10, Box 1, Folder 26)*

Instead of a “straightforward procedure for the definition of concepts,” in other words, operationism predominantly functioned as a new weapon to continue existing theoretical rivalries by different means. Whereas Boring and his students reduced psychology to the study of discriminatory capacities, defining operations as discriminations, Skinner believed that a completely different set of concepts was needed. For him, a true operationist analysis would show that the concepts of traditional psychology are confused, just as his own analysis had shown about the conventional notion of “reflex.”

Considering these developments, it is not surprising that Bridgman soon became fed up with the way in which the Harvard psychologists used his appeal for conceptual hygiene. Indeed, when Stevens had the opportunity to work with Bridgman for a semester after he had been awarded a General Education Board Training Fellowship (May 23, 1935, HUGFP 2.10, Box 1, Folder 8), it soon became clear that an unbridgeable gap had emerged. In a letter to his close colleague Arthur Bentley, Bridgman complained that Stevens' version of operationism was “just plain twisted” and by May 1936, he said that he had “washed [his] hands of him” (Bridgman to Bentley, May 4, 1936, HUGFP 4234.10, Box 1, Folder 10).^28^ In the 8 years between *The Logic of Modern Physics* and Stevens' “The Operational Basis of Psychology,” in other words, the psychologists' ideas about operational definition had been so adapted to their own theoretical needs that, for Bridgman, they had evolved beyond recognition.

## Endnotes

1 In general, operationism seems to have been “in the air” at the time. See Rogers (1989) for a reconstruction of the historical and contextual factors that contributed to the success of operationism in the 1930s and 1940s. For an overview of the intellectual climate at Harvard, see Isaac (2012).

2 The archival evidence on which this paper is built consists primarily of material from the E. G. Boring Papers (collection HUG 4229.5), the P. W. Bridgman Papers (collection HUG 4234.xx), the B. F. Skinner Papers (collection HUGFP 60.xx as well as accession 14328), and the S. S. Stevens Papers (collection HUGFP 2.xx). Documents from these collections will be referred to by collection code, date of creation (if known), and box, folder, and/or item number. In transcribing autograph notes, drafts, and letters, I have aimed to minimize editorial interference and chosen not to correct ungrammatical shorthand.

3 The remainder of this section is based on Verhaegh (2020).

4 Bridgman believed that we cannot *define* what an operational analysis is but that we can only see what the operational point of view entails by applying it to specific concepts. See, for example, Bridgman's letter to Hornell Hart: “I have never given a formal definition of what the operation is. This has been deliberate on my part. I have always insisted […] that the best way of learning what the operational approach is, is to see it in action as applied to concrete examples” (May 27, 1953, HUG 4234.10, Box 3, Folder 8).

5 The remainder of this section is largely built on Verhaegh (2019).

6 See Smith (1986, ch. 9) for the reconstruction of Mach's influence on Skinner's early theoretical development.

7 See also Skinner (1979, p. 115).

8 As an example, Boring discusses Watson's proposal to include verbal reports as explicit responses, thereby assimilating all the results of introspection to behaviorism.

9 Recall also Skinner's description of his first aim in the above‐discussed 30‐year “plan of the campaign”: “Experimental description of behavior […] *No surrender to the physiology of the central nervous system*” (November 17, 1932, HUGFP 60.50, Box 3, Folder 6, my emphasis).

10 See also Skinner (1979, pp. 93–94). Skinner's antipathy toward Boring is a constant in the early 1930s. See, for example, Skinner (1979, p. 94): “Boring developed the theme […] in a book […] which I used for years as an instrument of self‐management. Whenever I found myself losing interest in the work at hand or even simply feeling tired, a few pages of Boring's book had the effect of a dozen cups of coffee. What infuriated me was his refusal to recognize the possibility of a science of behavior.” Skinner here refers to Boring's *The Physical Dimensions of Consciousness* (1933), which will be discussed in Section 5.

11 Some of these passages are also quoted in Skinner (1979, pp. 72–73) and Coleman (1985).

12 Boring was not the only psychologist who did not understand Skinner's aims. Paul Huston, a former fellow graduate student, reported that people at Northampton were discussing the paper but “were a little puzzled” because it did not mention any experiment (November 5, 1931, HUGFP 60.10, box 1). Similarly, Don Purdy noted that he was “not so clear as to just what” the paper had “to say regarding the causal theory of behavior” (November 5, 1931, HUGFP, 60.10, box 1). See Verhaegh (2019).

13 I thank Julie Vargas for helping me transcribe Skinner's marginalia on Boring's comments.

14 Coleman (1985) rightly suggests that Skinner's thesis also appears to have offended Boring as a historian. Indeed, certain passages from Boring's letter suggest that he was also upset about Skinner's selective and uncritical history of the notion of reflex.

15 I call Boring's position *proto*‐operationism because it predates the publication of Bridgman's *The Logic of Modern Physics*.

16 See also Rogers (1989, p. 146), Green (1992, p. 297), and Feest (2005, p. 138).

17 Indeed, in looking back on his own operationist turn, Boring (1952, p. 44) himself also claimed that “his basic faith in operationism” was already “[t]ucked away” in his “little book” *The Physical Dimensions of Consciousness*.

18 It is not completely clear how Boring learned about Bridgman's operational approach. After all, Boring's dismissal of Skinner's approach strongly suggests that he did not yet know about Bridgman's book in 1930 (or, at the very least, that he failed to see the connection between Bridgman's approach and Skinner's project). Perhaps Bridgman himself indirectly played an important role in convincing Boring to discuss the operational approach in the seminar. For both Bridgman and Boring were active members of the Harvard Shop Club, a small society in which Harvard professors presented and discussed their work. See, for example, Boring's invitation to Bridgman from October 8, 1931 (HUG 4229.5, Box 5, Item 105).

19 See also Michell's (2004, p. 141) assessment in his *Measurement in Psychology*: “McGregor […] confined himself to psychophysics, thus not attending to intellectual abilities, personality traits, or other psychological attributes.”

20 Note that in defining psychophysical research in this way, McGregor implicitly circumvented Johnson's worry that psychophysics relies on a concept (“intensity of sensation”) that cannot be operationally defined (see Section 3). See, for example, McGregor's (1935, p. 263) discussion of Fechner's law, which concludes that γ is a magnitude “measured under special operational conditions which are specifically designed to shed light upon the functioning of the organism” and that γ is “as physical or as psychological” as β. See also “The Direct Estimation of Sensory Magnitudes: Loudness” (Stevens, 1956) for Stevens' implicit response to Johnson's argument. Unfortunately, McGregor and Stevens have never explicitly responded to Johnson's paper.

21 Michell (2004) even goes so far as to claim that McGregor's paper was “aged wine in [a] new operationist bottle” as his reinterpretation of psychophysics had already “been canvassed by Boring (1921) more than a decade before” (p. 142). This is too strong, however, as Boring did not yet fully endorse the “psychology of capacity” approach in 1921.

22 See, for example, Stevens (1968, p. 600).

23 See also the similarities between McGregor's (1935, pp. 262–263) and Stevens' (1935a, p. 329) discussions of Weber's laws.

24 During his first semester at Harvard, Stevens was still registered as a student at the School of *Education*.

25 See Hardcastle (1995, pp. 420–422): “It would be natural to identify E. G. Boring […] as a motive force behind Stevens' operationism. Stevens suggested as much in a tribute he wrote of Boring in 1968 […] Yet Stevens' attribution is in the end too generous […] Boring's comments […] record not the molding of a disciple, but a healthy ‘give and take' between a vigorous senior professor and his energetic and (likely over‐)confident student.”

26 See, for example, Boring in his set of comments on the first draft (“I have made the argument to you orally this afternoon”), Stevens' reply to Boring's comments on the second draft (“Our discussion of this afternoon convinced me that…”), and Boring's comments on Stevens' third draft (“Our discussion of the meaning of similarity is a case in point”) (HUGFP 2.10, Box 1, Folder 3).

27 At the symposium on operationism, Skinner hit back by reducing operationism to behaviorism, calling it “methodological behaviorism.” See Skinner (1945), Day (1983), and Moore (2013a, 2013b).

28 See also Walter (1990, p. 184).
